# Quality improvement project: improving the confidence of junior doctors to manage emergencies; Drs abc in an acute psychiatric setting

**DOI:** 10.1192/bjo.2021.531

**Published:** 2021-06-18

**Authors:** Ahrane Jayakumar, Wren Erin-Jones, Simon Edwards

**Affiliations:** 1 Gordon Hospital (CNWL); 2 St Mary's Hospital, CNWL; 3 Medical Director and Consultant Physician, Diggory Division, CNWL

## Abstract

**Aims:**

To improve the confidence and preparedness of junior doctors in managing medical or psychiatric emergencies when on call at an inpatient psychiatric unit.

**Background:**

Facilities for emergency care differ between acute medical and psychiatric units. Protocols for managing acutely deteriorating patients and those requiring immediate resuscitation differ across these organisations.

Managing medical emergencies can be stressful for all involved. Junior doctors rotate between services where the level of support varies depending on specialty and setting. For doctors who have worked in a setting where the minimum emergency response includes a Resuscitation team, moving to an environment with less support available is a challenge.

In our unit, the protocol following an urgent call is for the on-call doctor (who has access to basic resuscitation equipment) to attend and assess the need for paramedics and transfer to local hospital. Stress can be worsened by change of environment, change of expectation and concern about best management in new settings.

**Method:**

A cohort of junior doctors were recruited. Baseline assessment included rating their confidence level (scale 1- 10), listing common medical and psychiatric scenarios they had experienced and those they felt least confident managing. Over a period of 10 weeks, follow-up data was obtained. Interventions to improve confidence were assessed during this period, including a handbook and a teaching session on emergency medications. At the end of the project a wordcloud was created in response to the request to “choose 5 words to describe your feelings when called to an emergency”. Identified themes have been fed back to relevant senior staff and will form the basis of future projects.

**Result:**

The initial average confidence score improved from 4.9 to 9.2 and was sustained out to 14 weeks. According the word cloud the most commonly used words were “morale” and “education”.

**Conclusion:**

Prior to the study, confidence levels amongst the Junior Doctors was low. Introduction of the handbook and teaching session led to an improvement which was sustained. Key themes identified using a word cloud were “morale” and “education”.

For junior doctors moving from between services, different expectations and protocol for management of emergencies can influence confidence levels. Psychiatric units should be cognisant of these concerns and implement evidence-based intervention to support junior doctor confidence and improve quality of working experience.

